# Incidental Findings in POCUS: “Chance favors the prepared mind”

**DOI:** 10.24908/pocus.v7i1.15629

**Published:** 2022-04-21

**Authors:** Sara Obeid, Benjamin Galen, Trevor Jensen

**Affiliations:** 1 Division of Hospital Medicine, Department of Medicine, San Francisco VA Health System, University of California San Francisco San Francisco USA; 2 Division of Hospital Medicine, Department of Internal Medicine, Albert Einstein College of Medicine and Montefiore Medical Center USA; 3 Division of Hospital Medicine at UCSF Health, Department of Medicine, University of California San Francisco San Francisco USA

**Keywords:** POCUS, incidental findings

Point of Care Ultrasound (POCUS) has the potential to rapidly aide in diagnostic algorithms at the bedside, however POCUS users are often faced with the dilemma of appropriate management of incidental findings 1. Incidental findings in POCUS are defined as any indeterminate, benign, or potentially concerning finding found unexpectedly that is not related to the patient’s chief complaint 2. Increased use of POCUS has driven the increased discovery of incidental findings, with a reported frequency between 1.6% to 26% depending on the institution, frequency of documentation, and level of experience 1, 2. While many incidental findings are benign, some are not and benefit from follow-up. This raises important concerns regarding the need for systematic, evidence-based guidelines to ensure necessary follow-up while avoiding unnecessary additional imaging, patient anxiety and increased healthcare costs 1, 3. 

In some instances, incidental findings can lead to diagnoses that significantly alter patient treatments. In this issue of POCUS Journal, Melissa Bouwsema and Colin Bell report a case of a patient with a history of nephrolithiasis presenting with renal colic symptoms who was found to have both recurrent nephrolithiasis and an iliac artery aneurysm on POCUS exam 4. While it is difficult to know if the aneurysm was truly incidental or contributing to the patient’s presentation, this rare entity has a high mortality rate and the astute POCUS user made a life-saving diagnosis that could easily have been missed 5. 

Louis Pasteur is credited with the quote “chance favors the prepared mind.” In POCUS this would suggest that users should be trained to identify incidental findings and to triage their approach to managing them. Triage is required, especially when performing “contextualized scanning” and POCUS during emergencies 6. Yet operationalizing this practice is challenging, as it is hard to anticipate and manage the triage behaviors of providers who encounter a finding they are often neither looking for nor trained to evaluate. Lack of systematic, organized approaches to incidental findings with non-standard ultrasound views can lead to erroneous interpretation of acquired images and either under or over-referral to additional care. For instance, serious harm could have resulted if the aneurysm in Bouwsema and Bell’s case had been noted but not acted upon rapidly. On the other hand, in a similar theoretical case of abdominal pain where large para-aortic nodes were confused with the aorta and or aortic abdominal aneurysm, unnecessary imaging could have been done, resulting in increased health care costs and patient anxiety, which is of particular importance for vulnerable and marginalized patients who experience frequent interruptions in care 1, 2, 3, 7. Furthermore, incidental findings must be communicated to the patient and subsequent care team to ensure proper documentation and assessment 8; and failure to do this appropriately may lead to medical legal claims 9. 

Optimal management of incidental findings in all types of medical imaging / radiology requires concrete, evidence based, uniformly practiced protocols and standardized image acquisition to improve inter-rater reliability and cost-effective treatment as outlined by the American College of Radiology (ACR) and Fleischer Society 1, 10. The burden of uniform application of guidelines for both practitioners and trainees falls on each individual institution and requires targeted training and ongoing discussion 11. While the case by Melissa Bouwsema and Colin Bell in this issue of POCUS Journal demonstrated a clear benefit to recognizing an unexpected or potentially incidental finding, developing a robust system to manage all types of incidental findings in POCUS is complicated. Further study of incidental findings by POCUS are required to inform guidelines in this area. 
